# The Spectral Properties of (-)-Epigallocatechin 3-O-Gallate (EGCG) Fluorescence in Different Solvents: Dependence on Solvent Polarity

**DOI:** 10.1371/journal.pone.0079834

**Published:** 2013-11-22

**Authors:** Vladislav Snitsarev, Michael N. Young, Ross M. S. Miller, David P. Rotella

**Affiliations:** 1 Montclair State University, Department of Biology and Molecular Biology, Montclair, New Jersey, United States of America; 2 Montclair State University, Department of Chemistry and Biochemistry, Montclair, New Jersey, United States of America; 3 Margaret & Herman Sokol Institute for Pharmaceutical Life Sciences, Montclair, New Jersey, United States of America; University of Quebec at Trois-Rivieres, Canada

## Abstract

(-)-Epigallocatechin 3-*O*-gallate (EGCG) a molecule found in green tea and known for a plethora of bioactive properties is an inhibitor of heat shock protein 90 (HSP90), a protein of interest as a target for cancer and neuroprotection. Determination of the spectral properties of EGCG fluorescence in environments similar to those of binding sites found in proteins provides an important tool to directly study protein-EGCG interactions. The goal of this study is to examine the spectral properties of EGCG fluorescence in an aqueous buffer (AB) at pH=7.0, acetonitrile (AN) (a polar aprotic solvent), dimethylsulfoxide (DMSO) (a polar aprotic solvent), and ethanol (EtOH) (a polar protic solvent). We demonstrate that EGCG is a highly fluorescent molecule when excited at approximately 275 nm with emission maxima between 350 and 400 nm depending on solvent. Another smaller excitation peak was found when EGCG is excited at approximately 235 nm with maximum emission between 340 and 400 nm. We found that the fluorescence intensity (FI) of EGCG in AB at pH=7.0 is significantly quenched, and that it is about 85 times higher in an aprotic solvent DMSO. The Stokes shifts of EGCG fluorescence were determined by solvent polarity. In addition, while the emission maxima of EGCG fluorescence in AB, DMSO, and EtOH follow the Lippert-Mataga equation, its fluorescence in AN points to non-specific solvent effects on EGCG fluorescence. We conclude that significant solvent-dependent changes in both fluorescence intensity and fluorescence emission shifts can be effectively used to distinguish EGCG in aqueous solutions from EGCG in environments of different polarity, and, thus, can be used to study specific EGCG binding to protein binding sites where the environment is often different from aqueous in terms of polarity.

## Introduction

**Figure 1:**
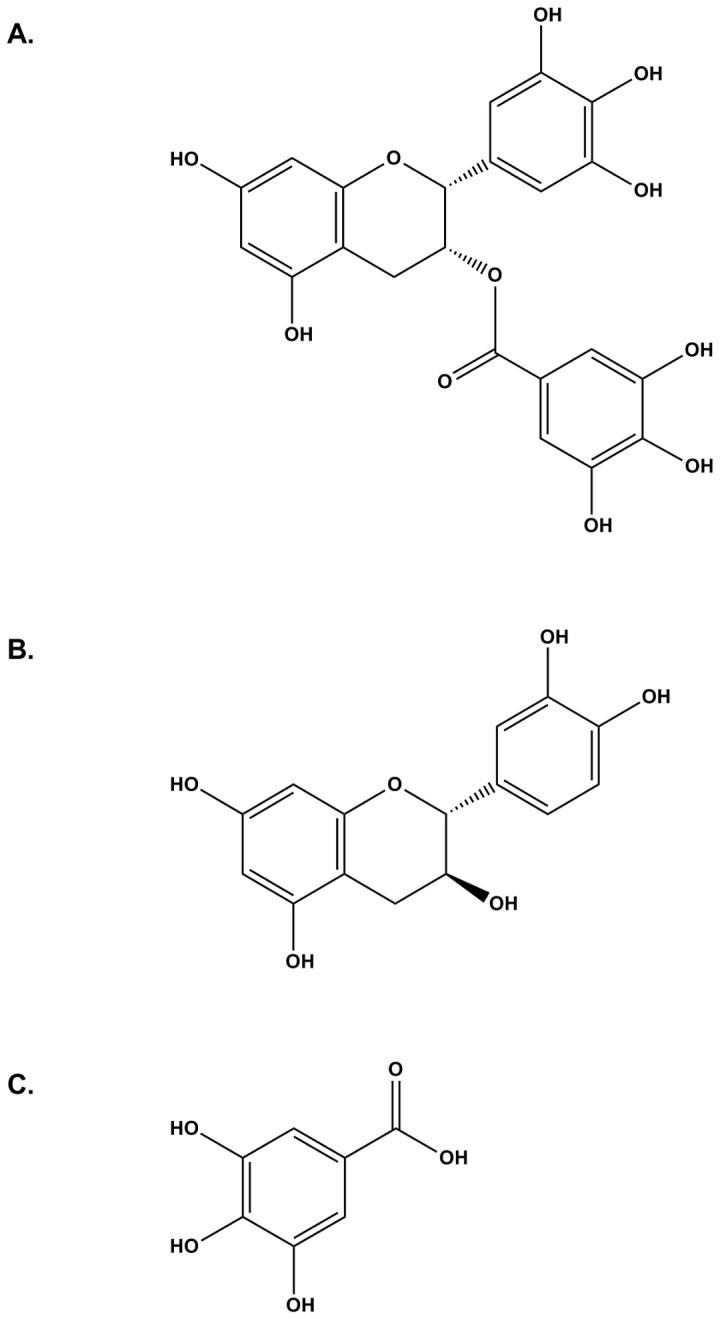
Structures of EGCG, catechin, and gallic acid. A. EGCG. B. Catechin. C. Gallic acid.

EGCG (Figure 1A), a major catechin in green tea, exhibits antioxidant [1,2], antimutagenic [3], anticancer [4-6], antiallergic [7,8], and antiatherosclerotic [9,10] properties. EGCG is carried by serum albumin[11] and has been identified as a novel inhibitor of heat shock protein 90 (HSP90)[12], a cytoplasmic chaperone protein, which has recently received much attention as a drug target for treatment of cancer [13,14]. As a chaperone protein, it stabilizes and maintains many client proteins and assists with normal protein folding and trafficking. These functions are essential in cell division and are being widely studied as a target for treatment of cancer[15]. To facilitate studies of the interaction of HSP90 with EGCG and analogs a direct binding assay would be useful and a frequently used very sensitive approach involves fluorescence spectroscopy. It is significantly more efficient to study binding of a ligand to a protein if the ligand is fluorescent using fluorescence polarization[16]. When excited at λ_Ex_=280 nm, catechin (Figure 1B), one portion of EGCG, has two fluorescence emission maxima, one peak at 314 nm and another peak ranging from 446 nm to 470 nm [17]. Another fragment of EGCG, gallic acid (Figure 1C), when excited at λ_Ex_=280 nm, has one fluorescence emission maximum ranging from 335 nm to 362 nm depending on the solvent[18]. Since these two fragments of EGCG are fluorescent, it is reasonable to hypothesize that EGCG is also fluorescent, and its fluorescence depends on the solvent. However, this hypothesis requires experimental verification since the two fragments when combined can quench each other’s fluorescence. We hypothesized that EGCG is fluorescent when excited at approximately λ_Ex_=280 nm and that its emission maxima are dependent on solvent. The EGCG fluorescence at the maximum of fluorescence excitation Ex_max_=331 nm/maximum of fluorescence emission Em_max_=455 nm or 550 nm was previously reported in a mixture of AN and aqueous solution significantly different from the cytoplasmic environment but solvent effects were not characterized [19]. In our study, however, it was found that EGCG fluoresces when excited at much shorter wavelengths. Here we report EGCG fluorescence in four solvents, 1) EtOH, a protic solvent, 2) AB at pH=7.0, as a model for the aqueous cytoplasmic environment, 3) DMSO, an aprotic polar solvent widely used for solubilization of water-insoluble organic compounds in biomedical research, and 4) AN, an aprotic solvent widely used for liquid chromatography characterization of organic molecules. The rationale for this choice of solvents is that binding EGCG to a protein such as HSP90[12] or to serum albumin[11] is likely to result in transition of EGCG from a predominantly aqueous environment to a less polar milieu which may result in dramatic changes in fluorescence[20]. Being able to distinguish EGCG in these environments would provide an important tool for studying EGCG binding to proteins and offer the possibility of a direct binding assay using a target protein.

## Materials and Methods

Absorbance of EGCG was measured at 10 µM with Nanodrop ND-1000 spectrophotometer (Thermo Fisher Scientific Inc.) at room temperature and standardized for 1 cm lightpath according to the Nanodrop’s manual. Extinction (*ε*) s: ε=Absorbance/lightpath (cm)/concentration (M).

Fluorescence of EGCG was measured at 10 µM with Hitachi F-7000 spectrofluorometer (Hitachi High-Technologies Co.) in a 1 cm quartz cell thermostated at 20°C. The slit width was 5 nm, PMT voltage 700 V, scan speed 240 nm/min.

The absorbance and fluorescence spectra were exported to and plotted with Origin 9 software (OriginLab Co).

EGCG was prepared as a 30 mM stock in DMSO, aliquoted with an Eppendorf Repeater® plus at 2 µL to avoid decomposition due to the freeze-thaw cycling and stored at -20°C. Before experiments, 4 µL of DMSO were added to a vial to bring EGCG concentration to 10 mM before 1/1000 dilution to the experimental concentration of 10 µM. Spectral characteristics were measured at 10 µM in AN, an aprotic solvent, and in AB containing KCl (150 mM), HEPES (10 mM) intended to mimic the aqueous cellular environment and at pH=7.0. We chose to measure fluorescence in a pH-buffered AB at a neutral pH=7.0 rather than distilled deionized water because ambient CO_2_ makes pH uncertain that may affect fluorescence and EGCG stability. We started initial measurements in DMSO, an aprotic solvent and included AN since DMSO has a cut-off at 265 nm (determined as absorbance of 1.00 in a 1 cm cell vs. water) rendering measurements of absorbance at shorter wavelengths impossible. Spectral measurements in DMSO were done in 100% DMSO, while solutions in AN and AB contained 0.1% DMSO. Therefore, 0.1% DMSO was added to the AN and AB blanks. It is noteworthy that even 0.1% DMSO can present a problem for UV absorbance and fluorescence measurements because the cut-off of 0.1% DMSO in EtOH is 229 nm, and in AB is 223 nm (not shown).

All chemicals were purchased from Sigma-Aldrich Co. The purity and structure of EGCG were confirmed by NMR and LCMS. 18.2 MΩ water (Milli-Q, Millipore) was used for all experiments.

## Results

### EtOH

**Figure 2:**
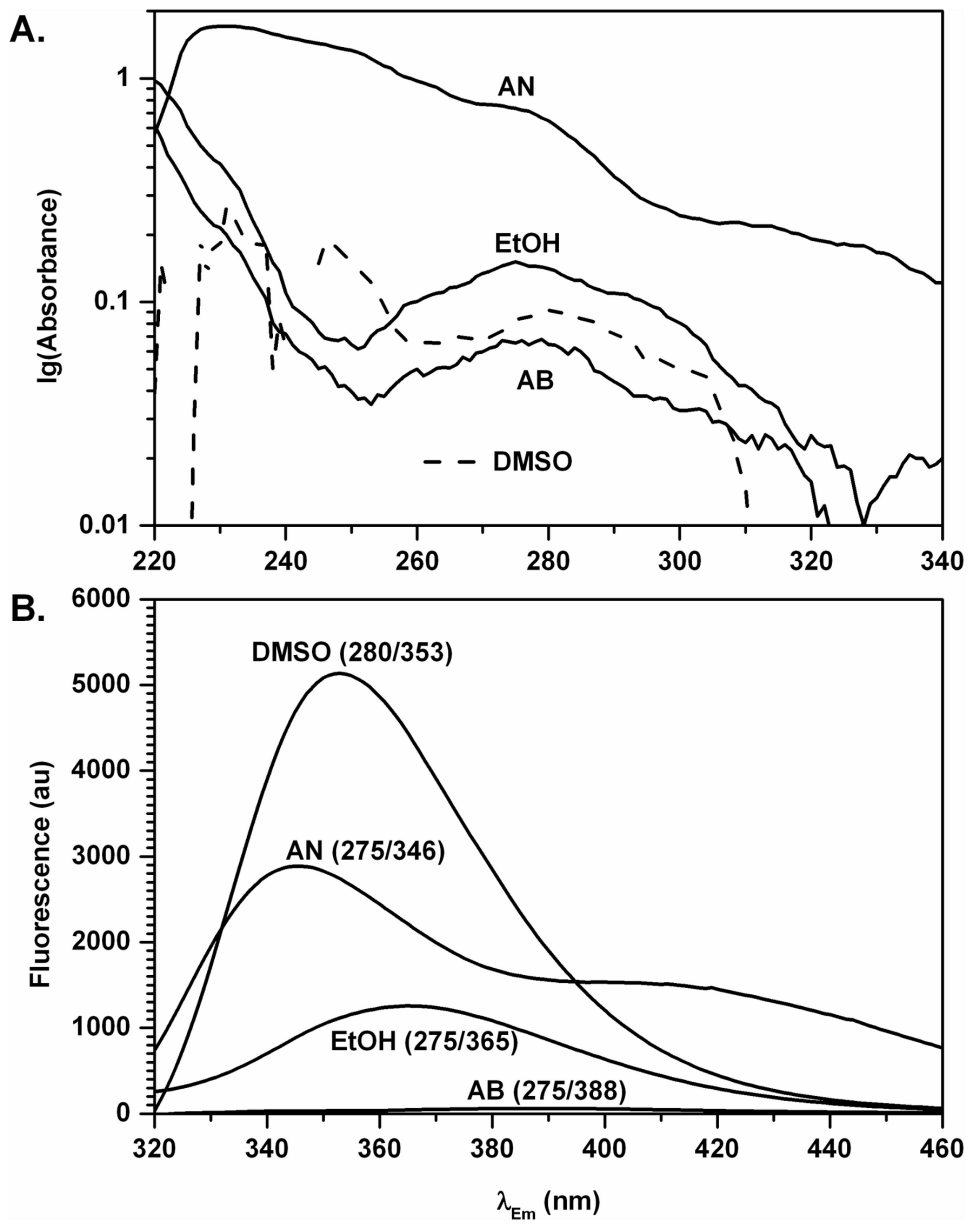
Spectra of EGCG in AB, AN, DMSO, and EtOH. A. Representative absorbance spectra of EGCG in acetonitrile (AN), ethanol (EtOH), dimethylsulfoxide (DMSO, dashed line for clarity), and aqueous buffer (AB). Absorbance is expressed in logarithmic units. B. Emission spectra of EGCG in dimethylsulfoxide (DMSO), acetonitrile (AN), ethanol (EtOH), and aqueous buffer (AB). Excitation wavelength/Em_max_ are indicated. All spectra were taken with the same spectrofluorometer settings.

Absorbance of 10 µM EGCG in EtOH at the wavelength of maximum UV absorbance UV_max_=275 nm was 0.151±0.004 (n=4), 0.175±0.017 (n=4), and at UV_max_=277 nm it was 0.138±0.014 (n=4) in three independent experiments (Table 1, Fig. 2A). Monograph #3526 of the Merck Index 14^th^ reports extinction ε=11,500 (cm^-1^M^-1^) at UV_max_=275 nm. When excited at UV_max_=275 nm, the intensity of fluorescence was 1,258 arbitrary units (au) at Em_max_=365 nm (emission scan) (Figure 2B). An additional 3D fluorescence scan with the excitation from λ=220 to 300 nm, and emission from λ=320 to 400 nm detected a larger peak at Ex_max_=275 nm/Em_max_=365 nm with FI= 1,495 au and a smaller peak at Ex_max_=235 nm/Em_max_=373 nm with FI= 389 au (Table 1).

**Table 1:** Spectral properties of EGCG.

	**AB**	**AN**	**EtOH**	**DMSO**
**UV_max_ (*ε*)**	275 nm (6,600) 270 nm (10,000) 276 nm (8,700) **274±3 nm (8,400±1,700**)	231 nm (171,200)	275 nm (15,100) 275 nm (17,500) 277 nm (13,800) **276±1 nm (15,000±2,000**)	280 nm (9,200) 280 nm (12,100) 279 nm (9,500) **280±1 nm (10,000±2,000**)
**Ex (nm)/Em_max_ (nm): FI (au)**	275/388:60.4 280/389:28.0^a^ 237/396:18.5^a^	275/346:2,888^b^ 275/402:1,535^b^ 272/343:3,021^c^ ~231/344:400^c^	275/365:1,258 275/365:1,495^d^ 235/373:389^d^	280/353:5,137 276/351:5,599

**AB**, aqueous buffer; **AN**, acetonitrile; **EtOH**, ethanol; **DMSO**, dimethylsulfoxide; **UV_max_**, the wavelength of maximum UV absorbance; **ε**, calculated extinction of absorbance; **UV_max_** and **ε**mean values±standard deviations (Mean±SD) of three independent experiments are in bold; **Em_max_ (Ex**), the maximum of fluorescence emission excited at a given λ=Ex, **FI**, fluorescence intensity, expressed as au (arbitrary units). The solvents in the tables are listed in the order of decreasing **∆*f***, orientation polarizability[20,21]. ^a-d^ Data obtained from the same scans are superscripted by the same letter.

### AB

Absorbance of 10 µM EGCG was 0.066±0.004 (n=4) in AB at UV_max_=275 nm, 0.100±0.005 (n=4) at UV_max_=270 nm, and 0.087±0.007 (n=4) at UV_max_=276 nm in three independent experiments (Table 1, Figure 2A). When excited at UV_max_=275 nm, the intensity of fluorescence was 60.4 au at Em_max_=388 nm (Figure 2B, dotted line; Table 1). The 3D fluorescence scan with the excitation from λ=220 to 300 nm, and emission from λ=320 to 400 nm detected a peak at Ex_max_=280 nm and Em_max_=389 nm (Table 1). Interestingly, similar to EtOH, another smaller peak was detected at Ex_max_=237 nm/Em_max_=396 nm with FI= 18.5 au (Table 1).

### DMSO

Absorbance of 10 µM EGCG in DMSO at UV_max_=280 nm was 0.092±0.008 (n=6), 0.121±0.009 (n=4), and at UV_max_=279 nm it was 0.095±0.008 (n=4) in three independent experiments (Figure 2A, dotted line; Table 1). When excited at UV_max_=280 nm, the intensity of fluorescence was 5,137 au at Em_max_=353 nm (Figure 2B; Table 1). Additional 3D fluorescence scan with the excitation from λ=220 to 300 nm, and emission from λ=320 to 400 nm revealed a peak at Ex_max_=276 nm/Em_max_=351 nm with FI=5,599 au. A smaller peak at λ_Ex_~235-237 nm was detected with the 3D scans in both EtOH and AB but this smaller peak, if existed, could not be detected in DMSO due to the cut-off at 265 nm (see Methods).

### AN

The inability to detect EGCG fluorescence in DMSO at λ_Ex_~235-237 nm due to the cut-off effects of DMSO (an aprotic solvent) to contrast with protic solvents such as AB and EtOH, made us use AN, a non-hydrogen bonding polar solvent with a cut-off of 190 nm similar to water (191 nm). Absorbance of 10 µM EGCG in AN at UV_max_=231 nm was 1.712±0.004 (n=4) (Figure 2A, Table 1). For comparison with other solvents used, the emission spectrum of EGCG in AN was measured at λ_Ex_=275 nm (Figure 2B), and two distinct emission peaks were detected, the larger peak being at λ346 nm and the smaller and wider peak being at approximately λ400 nm. A 3D fluorescence scan with the excitation from λ200 to 290 nm, and emission from λ300 to 350 nm detected a peak at Ex_max_=272 nm/Em_max_=343 nm with FI=3,021 au. Interestingly, this peak dominated over another small peak with Ex_max_~231 nm and Em_max_~344 nm with FI~400 au which could be separated only computationally (Table 1).

### Lippert plot

**Figure 3:**
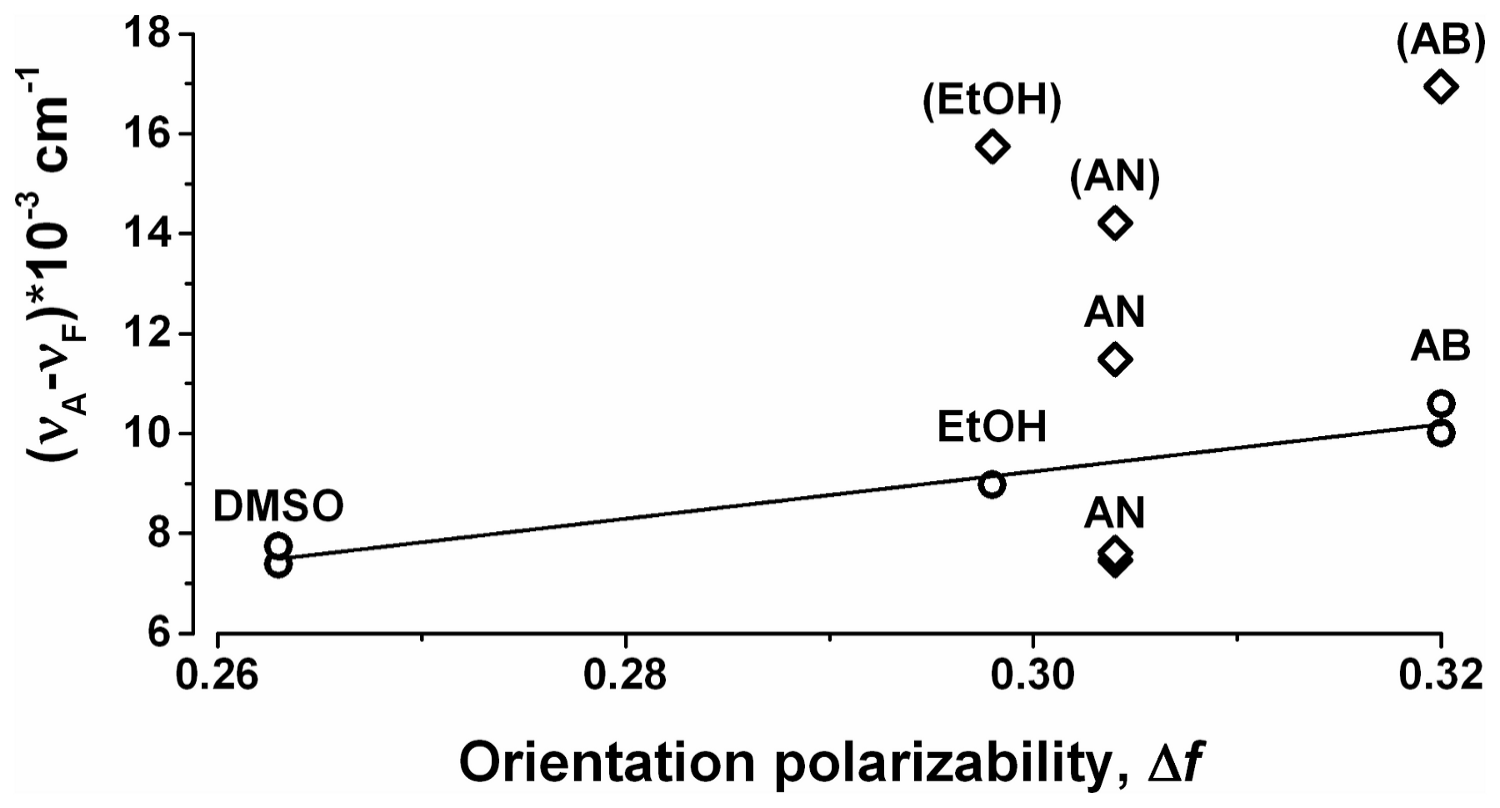
Lippert plot (see Table 2 and DISCUSSION for explanations). The Stokes shift (ν_A_- ν_F_)*10^-3^ (cm^-1^) was plotted against the orientation polarizability Δ*f* for the different solvents from Table 1 according to the following calculation: (ν_A_- ν_F_)*10^-3^ (cm^-1^) = 10^4^/Ex(nm) - 10^4^/Em_max_(nm) (Table 2). The labels for the smaller excitation peaks are indicated in the parentheses. The larger fluorescence maxima, which follow the Lippert-Mataga relation are indicated with open circles (), and are fitted with the linear regression (adjusted R^2^=0.95).

The Stokes shift (ν_A_- ν_F_)*10^-3^ (cm^-1^) was plotted against the orientation polarizability Δ*f* for the different solvents from Table 1 to build a Lippert plot[20] (Figure 3) according to the following calculation: (ν_A_- ν_F_)*10^-3^ (cm^-1^) = 10^4^/Ex(nm) - 10^4^/Em_max_(nm) (Table 2). The larger emission peaks in AB, EtOH, and DMSO are single emission peaks that follow the Lippert-Mataga equation[20] since they fall into almost perfect line with R^2^=0.95 (Figure 3, open circles).

**Table 2:** Solvent polarity and Stokes shifts of EGCG.

Solvent	Δ*f*	Ex	Em_max_	(vA-vF)*(10^-3^) cm^-1^	Symbols and labels in Figure 3
AB	0.32	275	388	10.6	** AB**
AB	0.32	280	389 ^a^	10.0	** AB**
AB	0.32	237	396 ^a^	16.9	** (AB)**
AN	0.30	275	346 ^b^	7.5	** AN**
AN	0.30	275	402 ^b^	11.5	** AN**
AN	0.30	272	343 ^c^	7.6	** AN**
AN	0.30	231	344 ^c^	14.2	** (AN)**
EtOH	0.30	275	365	9.0	** EtOH**
EtOH	0.30	275	365 ^d^	9.0	** EtOH**
EtOH	0.30	235	373 ^d^	15.7	** (EtOH)**
DMSO	0.26	280	353	7.4	** DMSO**
DMSO	0.26	276	351	7.7	** DMSO**

**AB**, aqueous buffer; **AN**, acetonitrile; **EtOH**, ethanol; **DMSO**, dimethylsulfoxide; **UV_max_**, the wavelength of maximum UV absorbance; **ε**, calculated extinction of absorbance; **UV_max_** and **ε**mean values±standard deviations (Mean±SD) of three independent experiments are in bold; **Em_max_ (Ex**), the maximum of fluorescence emission excited at a given λ=Ex, **FI**, fluorescence intensity, expressed as au (arbitrary units). The solvents in the tables are listed in the order of decreasing **∆*f***, orientation polarizability[20,21]. ^a-d^ Data obtained from the same scans are superscripted by the same letter.

## Discussion

### Two excitation maxima of EGCG

Two excitation maxima of EGCG fluorescence were found in AB, EtOH, and AN. One smaller peak can be observed when EGCG is excited at approximately 235 nm with Em_max_ at 396 nm in AB, ~344 nm in AN, and 373 nm in EtOH (Table 1). In DMSO, the smaller peak cannot be distinguished due to high absorbance of DMSO at wavelengths shorter than 265 nm. Another single peak of much higher emission intensity compared to the smaller peak for each given solvent was found when EGCG is excited between 275 and 280 nm with Em_max_ between 350 and 390 nm in AB, EtOH, and DMSO (Table 1, Figure 2B). The distinct maxima of the fluorescence excitation in all solvents tested point to two distinct dipoles in EGCG and are important for further characterization of the UV spectra of EGCG and its derivatives. In the following discussion, we pay more attention to the larger peaks because 1) their higher fluorescence intensities are more practical for EGCG-protein binding studies, and 2) the shorter excitation wavelengths of the smaller peaks are impractical due to the cut-off properties of many organic solvents, as DMSO shows in our case.

### Emission maxima of EGCG depend on solvent polarity

The emission peak in AB shifted to a longer wavelength compared to EtOH (Table 1, Figure 2B). This corresponds with the increased polarity of water compared to EtOH (orientation polarizability ∆*f*=0.320 and 0.298, respectively [20,21], Table 2). The intensity of the fluorescence in AB was significantly quenched compared to EtOH (Table 1, Figure 2B). We did not elucidate the exact mechanism of this quench. DMSO has a smaller orientation polarizability ∆*f* of 0.263 [20,21] compared to AB and EtOH (Table 2) and a smaller Stokes shift (Table 1, Figure 2B). This is in a good agreement with solvent polarity and the fluorescence emission shift [20]. The fact that the fluorescence intensity in EtOH, a protic solvent, is only approximately one fourth of that in DMSO, an aprotic solvent, may argue in favor of the H^+^-dependent quench of fluorescence in AB. In AN, excitation at 275 nm resulted in two distinct emission maxima (Table 1, Figure 2B) suggesting non-specific solvent effects on EGCG fluorescence. Together with the fact that EGCG fluorescence in AN does not follow the Lippert equation (Figure 3), it indicates that at least two electronically distinct species may be formed due to interaction of EGCG and AN.

### Stokes shifts of the larger fluorescence peaks in AB, EtOH, and DMSO follow Lippert-Mataga equation

We found that Stokes shifts of EGCG fluorescence depend on solvent polarity (Table 2, Figure 3). Stokes shifts of the larger fluorescence peaks in AB, EtOH, and DMSO (but not AN) follow the Lippert-Mataga relation (Figure 3, open circles) since they fall into almost perfect line with R^2^=0.95 (Figure 3, open circles). If EGCG fluorescence in AN followed the Lippert-Mataga equation [20], one single emission maximum was found between 365 nm (Em_max_ in EtOH) and 388 nm (Em_max_ in AB) because orientation polarizability ∆*f* of AN (0.304) is between those of EtOH (0.298) and AB (0.320) [20,21] (Table 1). Interestingly, the Stokes shifts for two larger peaks in AN lie above and below the best linear fit for AB, EtOH, and DMSO at approximately the same distance. Additional theoretical and experimental investigation are necessary to explain if this observation is coincidental or follows natural law. Abnormal EGCG fluorescence in AN taken together with the fact that UV_max_ of EGCG in AN follows different pattern than UV_max_ of EGCG in AB, EtOH, and DMSO (Table 1, Figure 1A) points to non-specific AN effects on EGCG fluorescence. Thus, previously reported EGCG fluorescence at Ex_max_=331 nm/Em_max_=455 and 550 nm in a mixture of AN and aqueous solution with uncertain pH [19] is difficult to interpret.

We demonstrated that EGCG is a fluorescent molecule and, importantly, its fluorescence is significantly dependent on the polarity of solvent. Interaction of EGCG with a binding pocket of a protein is likely to transfer EGCG from aqueous environment to one with different polarity that is expected to significantly change fluorescence intensity and shift emission maxima. We suggest that both changes in fluorescence intensity and fluorescence emission shifts can be used to study interaction of EGCG with HSP90 or other proteins. In addition, high EGCG fluorescence is useful for studies of binding to proteins with fluorescence anisotropy approach [16].
